# Contact Pattern Recognition of a Flexible Tactile Sensor Based on the CNN-LSTM Fusion Algorithm

**DOI:** 10.3390/mi13071053

**Published:** 2022-06-30

**Authors:** Yang Song, Mingkun Li, Feilu Wang, Shanna Lv

**Affiliations:** 1 School of Electronic and Information Engineering, Anhui Jianzhu University, Hefei 230601, China; esunny@ahjzu.edu.cn (Y.S.); lmk@ahjzu.edu.cn (M.L.); shnaan@ahjzu.edu.cn (S.L.); 2 Key Laboratory of Building Information Acquisition and Measurement Control Technology, Anhui Jianzhu University, Hefei 230601, China

**Keywords:** flexible tactile sensor, contact pattern, recognition, convolutional neural network (CNN), long short-term memory (LSTM) network

## Abstract

Recognizing different contact patterns imposed on tactile sensors plays a very important role in human–machine interaction. In this paper, a flexible tactile sensor with great dynamic response characteristics is designed and manufactured based on polyvinylidene fluoride (PVDF) material. Four contact patterns (stroking, patting, kneading, and scratching) are applied to the tactile sensor, and time sequence data of the four contact patterns are collected. After that, a fusion model based on the convolutional neural network (CNN) and the long-short term memory (LSTM) neural network named CNN-LSTM is constructed. It is used to classify and recognize the four contact patterns loaded on the tactile sensor, and the recognition accuracies of the four patterns are 99.60%, 99.67%, 99.07%, and 99.40%, respectively. At last, a CNN model and a random forest (RF) algorithm model are constructed to recognize the four contact patterns based on the same dataset as those for the CNN-LSTM model. The average accuracies of the four contact patterns based on the CNN-LSTM, the CNN, and the RF algorithm are 99.43%, 96.67%, and 91.39%, respectively. All of the experimental results indicate that the CNN-LSTM constructed in this paper has very efficient performance in recognizing and classifying the contact patterns for the flexible tactile sensor.

## 1. Introduction

With the development of microsensors and intelligent robot technology, tactile sensors have been receiving more and more attention. Recognizing different contact patterns applied to the tactile sensors plays a very important role in human–machine interaction. A flexible tactile sensor is an essential tactile information acquisition medium of the robot sensor system, and it has special advantages in detecting target surface texture and physical properties [1,2,3], which is conducive to establishing a more secure and reliable human–machine interaction system [4,5].

A flexible tactile sensor is a flexible device that senses the force on the surface. With its good flexibility and stretchability, it can be attached to the surface of irregular objects, and it is widely used in the research of robotic bionic skin [6,7,8,9]. Therefore, researchers improve the performance of sensors from the aspects of materials [10,11,12], multifunctional integration [13,14,15], and self-energy [16,17,18]. At present, flexible tactile sensors based on capacitive [19], piezoresistive [20,21], piezoelectric [22,23], and other mechanisms have been continuously proposed, and they have shown good application prospects. Among them, flexible tactile sensors based on piezoelectric mechanisms have high sensitivity and fast response ability [24], so they are widely used to detect dynamically changing tactile signals. In addition, piezoelectric tactile sensors can convert external mechanical pressure into electrical energy, which makes piezoelectric tactile sensors particularly important in self-powered applications.

Piezoelectric materials used to fabricate tactile sensors are mainly divided into inorganic piezoelectric materials, organic piezoelectric materials and their copolymers. On the one hand, inorganic piezoelectric materials such as PZT [25,26] hinder their application on complex curved surfaces due to their lack of flexibility. On the other hand, the organic piezoelectric material represented by polyvinylidene fluoride (PVDF) and its copolymer shows good flexibility, stable chemical properties, and low cost [27], so PVDF piezoelectric material is suitable for dynamic tactile perception. Luo et al. [28]. designed a capacitive sensor with a micropillar-polyvinylidene fluoride (PVDF) dielectric layer, which can achieve high sensitivity (0.43 kPa^−1^) at low pressure (<1 kPa). The 8 × 8 sensor array prepared based on the flexible tactile sensor can map the geometric shape of the pressing object by detecting the change of capacitance. Tang et al. [29]. proposed a piezoresistive sensor with good overall performance (response time less than 75 ms and sensitivity of −0.19 kPa^−1^) by using one-step thermal foaming technology to prepare a dome pattern and coating high conductivity graphene on its surface, which can be successfully applied in pulse detection and gait analysis. Ryu et al. [30] fabricated a self-powered flexible tactile sensor with an energy density of $4.3\;\mathrm{mW}/m^{2}$ through PVDF-Bi_4_Ti_3_O_12_ material, which can detect different human actions without an external power supply. Yi et al. [31] proposed a method for identifying the tactile surface roughness of a bionic fingertip, and the highest recognition accuracy of the collected pulse sequence based on the KNN algorithm reached (77.6±13.7)%. Qin et al. [32] realized the acquisition of surface topography signals based on PVDF bionic tactile sensors and used extreme learning machine instead of KNN or SVM to identify the roughness of the object surface, increasing the recognition accuracy to 97.88%. Gastaldo et al. [33] demonstrated PVDF piezoelectric tactile sensor to collect different contacts and designed a new tensor-based machine learning method to classify three types of contacts, with the highest accuracy of 78.3%.

The above analysis implies that piezoelectric tactile sensors are suitable for collecting tactile signals in the process of dynamic contact. Therefore, we hope to quickly prepare an intelligent, flexible tactile sensor with good flexibility and response speed through PVDF piezoelectric materials, which can be used to accurately detect and recognize various contact patterns.

To demonstrate this possible development trend, in this work, we exhibit a flexible tactile sensor with good flexibility, high sensitivity (10.53 mV/N), fast response rate (<3.2 ms), low hysteresis error (5.88%), and great repeatability error (3.42%) is proposed and fabricated, and the sensor is used to collect the tactile signals of stroking, patting, kneading, and scratching patterns. On this basis, the convolutional neural network (CNN) and the long short-term memory (LSTM) neural network are fused. The obtained CNN-LSTM fusion neural network has unique advantages in feature extraction and pattern recognition, and the recognition accuracy of the four tactile signals is 99.43%. The recognition results of the CNN-LSTM model are compared with the CNN and random forest (RF) algorithm, and the results show that the CNN-LSTM model can be more effectively applied to the recognition of tactile information.

## 2. Structure Design and Fabrication

### 2.1. Structure Design of the Sensor

**Figure 1:**
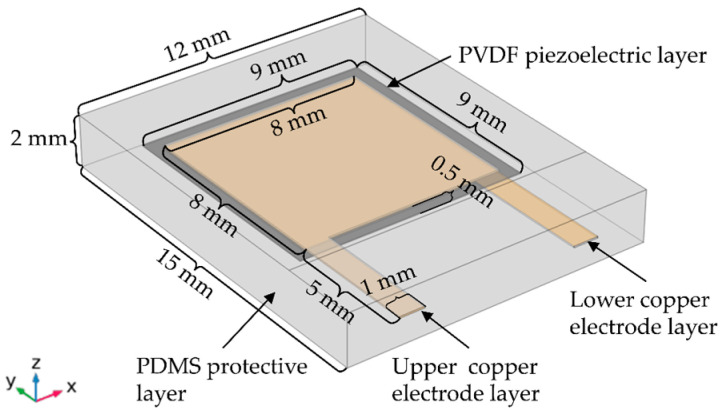
Structure of the flexible tactile sensor.

**Figure 2:**
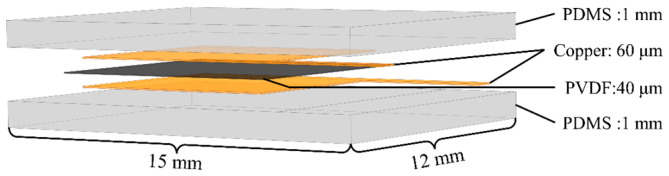
Schematic of the flexible tactile sensor with different components and layers.

In order to simulate the tactile function of human skin, we had tried to construct several structures with different shapes and sizes. After many simulations and careful analysis, we found that the simulation results of those structures are not as good as the structure designed in Figure 1. In Figure 1, a flexible piezoelectric tactile sensor is designed based on polyvinylidene fluoride (PVDF) material with high sensitivity, good flexibility, and high piezoelectric coefficient. Its structure mainly consists of a piezoelectric layer, two copper electrode layers, and a protective layer. The piezoelectric layer is a PVDF film with the dimension of 9 mm×9 mm×0.04 mm, which is composed of a PVDF polarization film and two silver electrode layers. It can convert pressure into an electrical signal to output. The copper electrode layers are composed of a double-layer “P”-type copper electrode consisting of an area of 8 mm ×8 mm and another area of 5 mm ×1 mm, and its thickness is 0.06 mm. Each copper electrode (the yellow part in Figure 1) is bonded with the PVDF piezoelectric film by conductive silver glue, and the PVDF film in the middle between the double-layer copper electrodes acts as the sensitive element of the sensor. The copper electrodes are located in the center of the PVDF film, and the vertical distance between the edge of the copper electrode and the edge of the PVDF film is 0.5 mm to prevent short-circuit from the PVDF piezoelectric film. The protective layer is made of polydimethylsiloxane (PDMS) with the dimension of 15 mm×12 mm×2 mm, which has good flexibility and can effectively transfer the pressure applied on the sensor to the sensitive element and protect the sensitive element inside the sensor from being damaged by the external environment. The schematic of the side view of the flexible tactile sensor with different components and layers is shown in Figure 2.

The properties of PDMS and PVDF are shown in Table 1. Table 1 indicates that PDMS has a low Young’s modulus, which makes the material much more flexible. The PDMS is used as the encapsulation layer of the tactile sensor, which endows the sensor with better flexibility. In addition, PDMS also has good biocompatibility and is very suitable to be used as the protective layer of flexible electronic skin. As the PVDF is a very thin film with a thickness of just 0.04 mm, it is also very flexible. The PVDF has a high piezoelectric coefficient and good flexibility, which is very suited for making sensitive elements of tactile sensors to obtain tactile information.

### 2.2. Simulation

**Figure 3:**
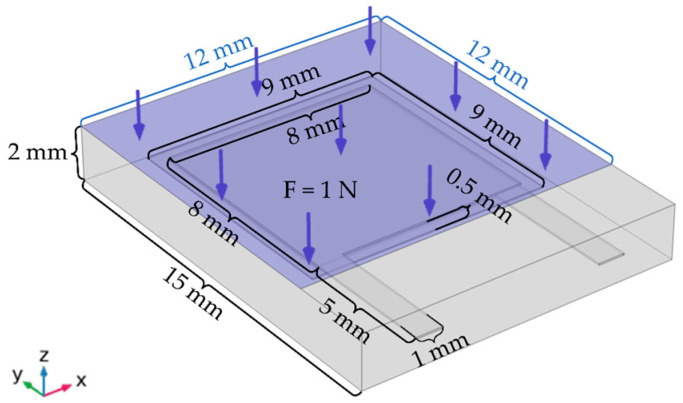
The schematic of the normal pressure applied to the sensor model.

**Figure 4:**
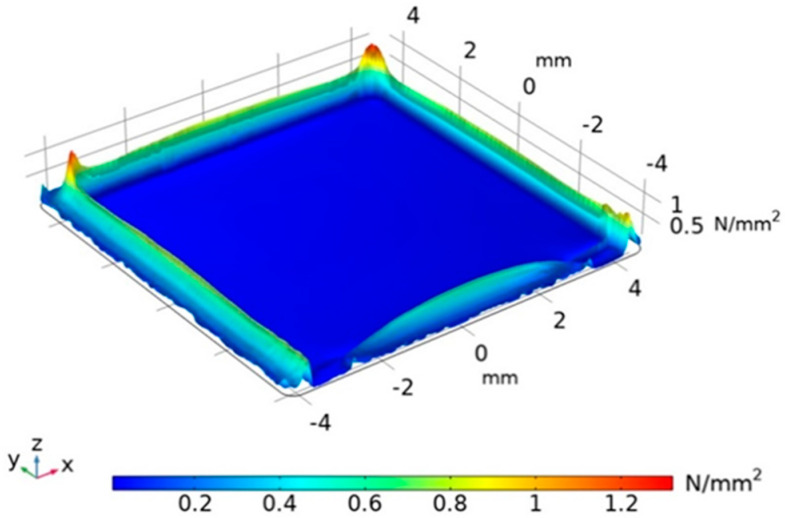
Stress distribution of the PVDF film.

Firstly, the multiphysics simulation software (COMSOL Multiphysics) is used to construct the geometric model of the flexible tactile sensor, as shown in Figure 3. Secondly, materials such as PVDF, PDMS, and copper are assigned to each domain of the geometric model constructed for the sensor. Thirdly, the solid mechanics field is added, and the boundary conditions of the sensor are constrained; in the interface of the boundary load, a total normal force of 1 N is applied to the upper surface of the sensor model and the area where the pressure applied is 12 mm × 12 mm; meanwhile, the lower surface of the sensor is fixed in the fixed constraint interface. Fourthly, the surface boundaries of the copper electrodes and PVDF are set as free quadrilateral meshes, respectively, and the swept meshes are divided along the thickness direction; then, the PDMS domain is meshed using the refined free tetrahedral mesh. Fifthly, the equations of physical field parameters are compiled and set in the steady-state study, and the equations are solved iteratively by the solver. Finally, the stress distribution on the PVDF film surface of the simulation results is drawn, as shown in Figure 4. The color shades shown in Figure 4 indicate different stress levels of the PVDF film.

After repeated stress experiments on the sensor model, the stress of the PVDF piezoelectric film covered by the copper electrode is almost uniformly distributed at $0.016\;N/{\mathrm{mm}}^{2}$, while the stress is obviously uneven within 0.5 mm of the edge of the PVDF film. The main reason for this phenomenon is that the hardness of the copper electrode and that of PDMS are different, and within 0.5 mm from the edge of the PVDF film, there is no copper covered. When the normal pressure is applied to the surface of the sensor, the PDMS and copper electrode transfer the pressure to the PVDF film; therefore, the stress of the PVDF film covered by the copper electrode is different from that of the PVDF film covered by the PDMS. The simulation results show that when PDMS is used to encapsulate the sensitive element, the stress distribution of the PVDF film covered by the copper electrode with the size of 8 mm×8 mm (dark blue area in Figure 4) is relatively uniform, and the sensor can convert the pressure into a stable electrical signal to output.

### 2.3. Fabrication of the Sensor

#### 2.3.1. Fabrication of the Sensitive Element

**Figure 5:**
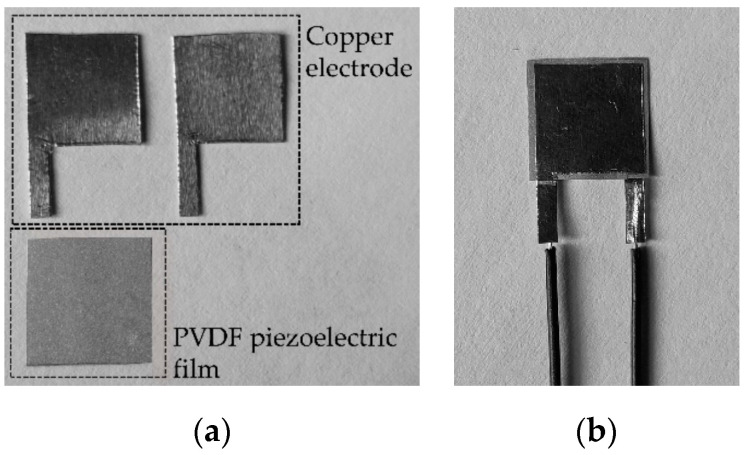
The preparation of the sensitive element: (**a**) component of the sensitive element; (**b**) sensitive element.

Firstly, the PVDF piezoelectric film (MEAS 1-1004346-0-M, TE Connectivity Ltd., Berwyn, PA, USA) with the dimension of 9 mm×9 mm×0.04 mm is made, as shown in Figure 5a. Secondly, the PVDF film is cleaned with propanol and deionized water, respectively, so as to corrode the metal burrs on the edge of the film. This step can prevent the PVDF film from short-circuiting along the thickness direction. Thirdly, the copper electrode is made into the “P” structure, as shown in Figure 5a, and the electrode consists of a square with the area of 8 mm ×8 mm and a rectangle with the area of 5 mm ×1 mm. After that, the conductive silver glue (SINWE 3703, Sinwe New material Co., Ltd, Shenzhen, China) is used to connect the copper electrode, the wire, and the PVDF piezoelectric film to form an overall sensitive element. In that step, the PVDF film is placed between the two P-structure copper electrodes, the two copper electrodes are located in the center of the PVDF film, and the vertical distance between the edge of the copper electrode and the edge of the PVDF film is 0.5 mm to prevent the PVDF piezoelectric film from short-circuiting. Then, the whole sensitive element is put into a drying oven (202-0A, Mingtu Machinery Equipment Co., Changge, China) at 65 ℃ for 4 h to reduce the contact resistance between the copper electrode and the PVDF film. Finally, the sensitive element of the sensor is obtained, as shown in Figure 5b.

#### 2.3.2. Encapsulation of the Sensitive Element

**Figure 6:**
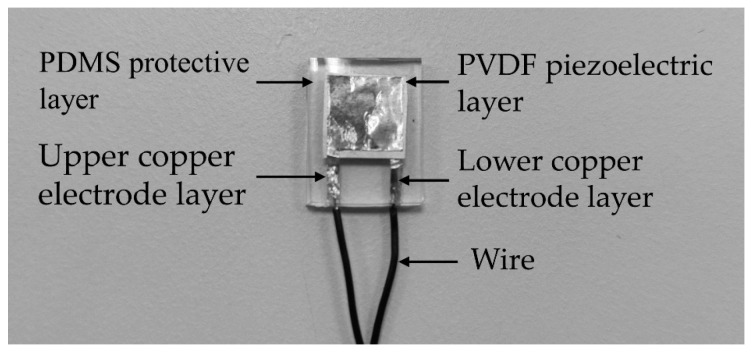
Prototype of the flexible tactile sensor.

As the sensitive element is gained, it should be encapsulated. The PDMS material is used as the protective and flexible component of the sensor to prevent the sensitive element from being damaged and worn. The encapsulation process of the sensitive element is as follows: step 1. PDMS and the curing agent (SYLGARD 184, Dow Corning Co., Midland, MI, USA) are poured into a beaker at a mass ratio of 10:1 and subjected to ultrasonic treatment for 20 min and magnetic stirring for 30 min, respectively, then a mixed solution can be gained; step 2. the solution is poured into a 15 mm ×12 mm ×5 mm glass vessel, and the height of the solution level is 1 mm, and then it is placed into a drying oven at 65 °C for 4 h to obtain the base element for the flexible tactile sensor; step 3. The sensitive element prepared, as shown in Figure 5b, is placed on the flexible base element in the glassware, and then the prepared solution is poured onto the sensitive element to obtain the PDMS protective layer whose thickness is 2 mm; step 4. the glassware containing the solution is placed into the drying oven at 65 °C for 4 h as the curing treatment, and then the encapsulated sensitive element can be taken away from the glassware, and the flexible tactile sensor prototype is obtained (shown in Figure 6).

## 3. Performance Testing and Data Acquisition

**Figure 7:**
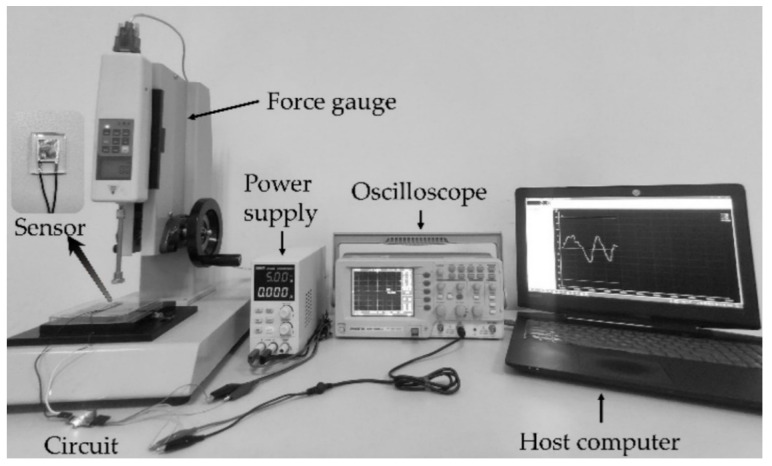
Experimental platform.

The experimental platform built in this paper mainly consists of a flexible tactile sensor, a force gauge (HP-500, Aidebao Instruments Co., Hangzhou, China), an oscilloscope (GDS-1052-U, GWINSTEK, Taiwan), etc., as shown in Figure 7. The sensor is fixed on the carrier of the force gauge shown in Figure 7, and the pressure loaded on the sensor could be converted into a voltage signal and output to the oscilloscope. To effectively filter out the interference of the 50 Hz power frequency signal, low-pass filtering is taken to reduce the noise of the output voltage signal, which can collect the pressure information loaded on the sensor more accurately. The calibration experiment and response time tests for the flexible tactile sensor are carried out, and the tactile signals loaded on the sensor are collected, which are the dataset for the contact pattern recognition.

### 3.1. Calibration Experiment

When a fixed pressure is applied to the piezoelectric tactile sensor slowly, there will be a phenomenon of charge leakage, which often leads to the inaccurate output of the voltage [34]. Therefore, in this paper, the relationship between the pressure loaded on the sensor and the corresponding output voltage is calibrated by fast unloading pressure. By unloading the pressure applied to the sensor quickly, the output voltage peak could be detected more accurately.

**Figure 8:**
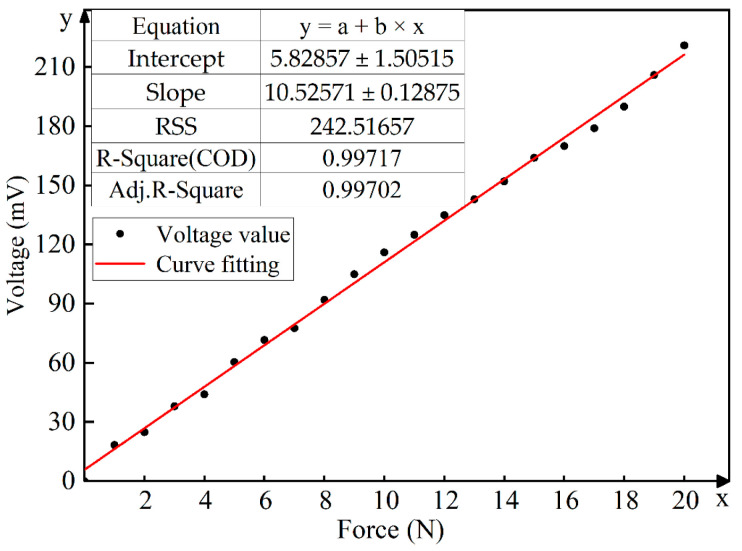
Fitting curve between input pressure and output voltage.

The pressure of 0 N to 20 N is applied to the tactile sensor with the step of 1 N, respectively. This process is repeated five times, and the corresponding peak value of the output voltage is saved when the pressure is unloaded each time. To reduce the influence of unloading speed on the peak value of output voltage, the average value of the five peak voltages is taken as the output voltage corresponding to the pressure loaded on the sensor. The relationship between the output voltage and the corresponding pressure is calibrated by the least square method, as shown in Figure 8.

As can be seen from Figure 8, the relationship between the pressure and the output voltage of the sensor is almost linear. With the range of 0–20 N, the fitting line between the pressure and the voltage is y=10.53x+5.83, which means that the sensitivity of the sensor is 10.53 mV/N, and the correlation coefficient is $R^{2}=0.997$.

### 3.2. Response Characteristic Testing

**Figure 9:**
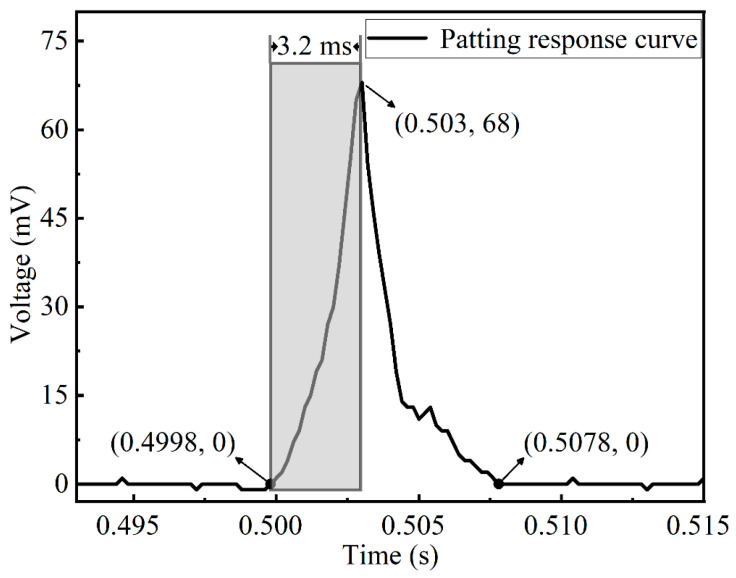
Voltage response curve of patting.

When an impact pressure is exerted on the surface of the sensor and quickly reaches the maximum value, the output charges of the sensor increased to the corresponding maximum value rapidly. The time difference in the appearance of the maximum values between the pressure and the output charges is the response time of the sensor [35]. By repeated experiments of four contact patterns (stroking, patting, kneading, and scratching) exerted on the sensor, the results show that the response time of the patting operation is 3.2 ms, which is the minimum among those of the four contact patterns. The voltage response curve of patting is shown in Figure 9.

Figure 9 shows that when a single patting operation applied to the sensor, the response voltage changed from 0.4998 s, and the value increased from 0 mV to 68 mV in 3.2 ms. Then, in the following 4.8 ms, the response voltage decreased from 68 mV to 0 mV. This indicates that the tactile sensor can quickly return to the initial state when stimulated by an external force. It has very good frequency response characteristics and can respond to the four contact patterns of stroking, patting, kneading, and scratching applied on the sensor surface in time.

### 3.3. Hysteresis Testing

Hysteresis is used to describe the extent to which the input and output characteristic curves do not coincide during forward and reverse strokes. The value of hysteresis could be computed as follows:

(1) $$\gamma_{H}=\frac{\Delta H_{max}}{Y_{FS}}\times 100\%$$

where $\Delta H_{max}$ represents the maximum output difference between the positive stroke and the negative stroke; $Y_{FS}$ is the full-scale output of the sensor.

**Figure 10:**
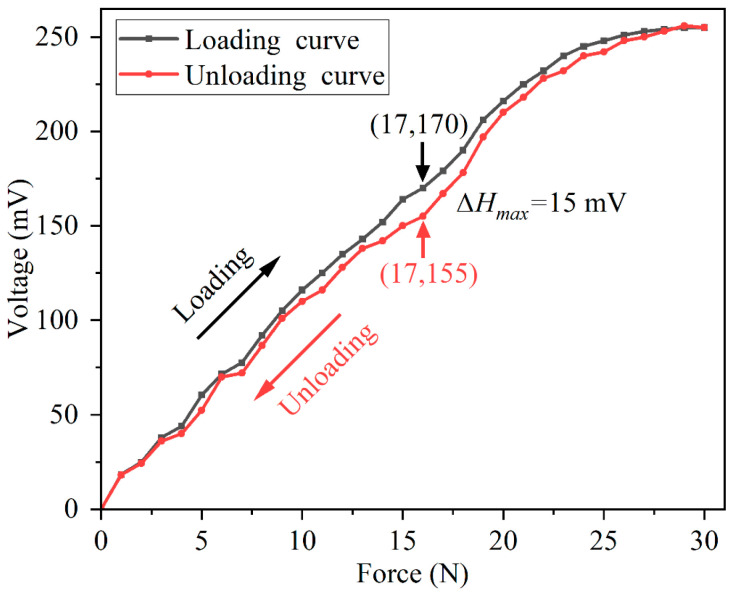
The hysteresis curves of the sensor.

In our work, the full-scale output is 255 mV, and the hysteresis curves of the sensor are shown in Figure 10. Figure 10 indicates that $\Delta H_{max}$ = ∣170 mV − 155 mV∣, so the $\gamma_{H}$ can be obtained as follows:

(2) $$\gamma_{H}=\frac{|170-155|}{255}\times 100\%=5.88\%$$

Figure 10 demonstrates that within the pressure range of 23−30 N, the sensitivity of the sensor decreases gradually, which means the upper limit of pressure that the sensor can detect is 30 N. The main reason is that with the increase in pressure, the amount of charge generated by the internal sensitive element PVDF tends to reach the saturation state gradually, and when the pressure is around 30 N, the output voltage of the sensor almost does not change.

### 3.4. Repeatability Testing

Repeatability refers to the degree to which the output characteristic curves of the sensor do not coincide when the input is loaded onto the sensor along the forward or reverse direction several times under the same conditions. The repeatability could be expressed as follows:

(3) $$\delta_{R}=\frac{|Z\cdot \delta_{max}|}{Y_{FS}}\times 100\%$$

where $\delta_{R}$ is the repeatability error. For a normal distribution, when *Z* equals 3, the confidence probability is 99.73%; $\delta_{max}$ represents the maximum standard deviation of all points on the actual measured curve. $Y_{FS}$ represents the full-scale output of the sensor.

**Figure 11:**
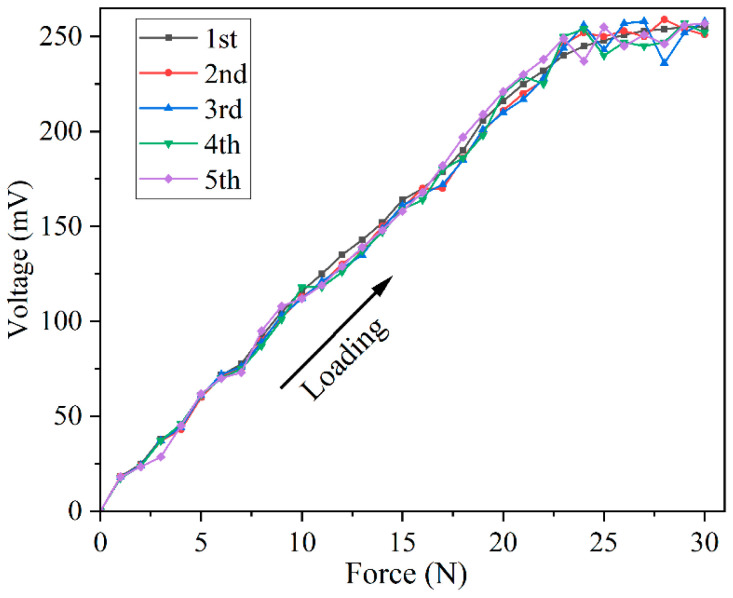
The repeatability curves of the sensor.

To ensure that the sensor can stably collect the accurate tactile information of the four contact patterns, the repeatability experiment of the sensor is conducted. In the experiment, the positive stroke pressure of 0–30 N is applied to the sensor with the step of 1 N, and the experiment is repeated five times, in turn, to obtain the repeatability curves, as shown in Figure 11. After calculation, it is gained that $\delta_{max}$ = 8.735, and then $\delta_{R}$ can be obtained as follows:

(4) $$\delta_{R}=\frac{|99.73\%\times 8.735|}{\frac{1}{5}\left(255+251+258+252+257\right)}\times 100\%=3.42\%$$

It can be seen from Figure 11 that the sensor has fairly good repeatability and stability when the pressure within 0–23 N is applied to the sensor. Within the pressure range of 23–30 N, the output voltage of the sensor tends to become saturated gradually, which the environment can easily disturb. Correspondingly, there exists a large deviation in the output voltage. Since the pressures of the four contact patterns applied to the sensor are all within the range of 0–23 N, the sensor could be used to collect tactile signals repeatedly.

The above results imply that the flexible tactile sensor not only has a very good linear relationship between the input pressure and the output voltage but also has a fast response rate, low hysteresis, and great repeatability and stability. It is suitable for the acquisition of tactile signals in the dynamic contact procedure.

### 3.5. Data Acquisition

In the experiment, there are four testers (two males and two females). Each tester applied four contact patterns of stroking, patting, kneading, and scratching to the flexible tactile sensor with any of his or her index fingers, respectively. When stroking, patting, or kneading was applied, the angle between the tester’s index finger and the upper surface of the sensor is about 45 degrees, and the contact area between the index finger and the sensor surface is about 10 mm × 10 mm. Scratching refers to rubbing the sensor surface with the index fingernail of the tester, and the index finger is perpendicular to the sensor surface during the rubbing process. During the experiment, the index finger should be kept clean and free of sweat so as to reduce the change in the friction coefficient between the finger and the sensor surface caused by the repeated application of the same operation.

**Figure 12:**
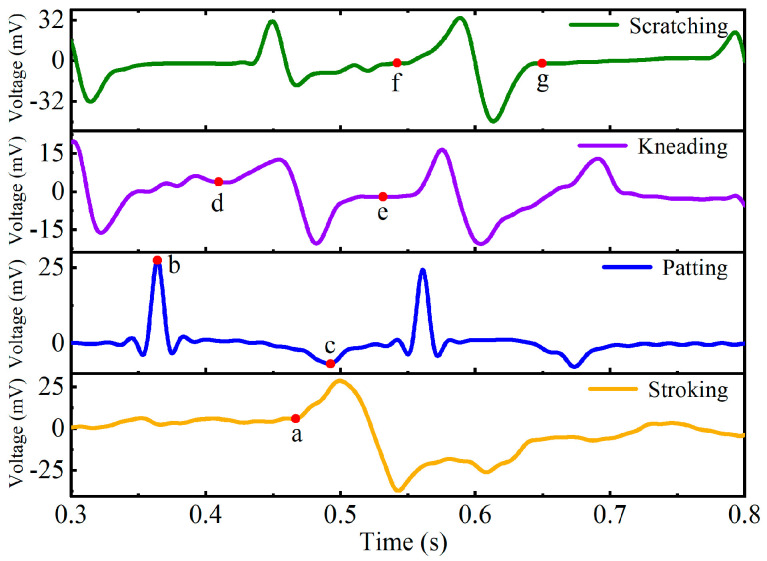
Voltage response curves of the four contact patterns.

When the contact patterns applied to the sensor, the corresponding voltage signal sequence generated by the four contact patterns are collected for each tester, respectively. In the experiment, the sampling frequency of the oscilloscope is 5 kHz, and 4000 time steps of voltage signals are collected within 0.8 s. The voltage response of the four contact patterns from 0.3 s to 0.8 s is shown in Figure 12. Due to the limitation of reaction time of the testers and the delay of data storage by the oscilloscope, the data characteristics of the voltage signals collected in the first 0.3 s are not obvious. Therefore, the response voltage curves of the four contact patterns shown in Figure 12 start from 0.3 s.

It can be seen from Figure 12 that when the tester stroked the sensor surface (the yellow curve in Figure 12), the sensor deformed by the contact pressure, and the response voltage increased gradually from point a. When the stroking pressure was removed, the sensor gradually returned to the initial state without deformation, and the response voltage returned to 0 mV correspondingly. In the experiment, single patting, two-consecutive patting, and three-consecutive patting are carried out on the sensor, respectively, and the results show that the characteristics of their voltage response curves are very similar. Therefore, Figure 12 only demonstrates the typical pattern curve of the two-consecutive patting (the blue curve in Figure 12). In the process of applying patting action to the sensor, the output peak voltage is at point b. When removing the patting pressure, the sensor returns to its initial state, and the minimum output voltage is at point c. When the index finger pulp of the tester is gently kneading on the sensor surface back and forth, the corresponding output voltage changes alternately between positive and negative (from point d to point e of the purple curve in Figure 12), and its voltage amplitude is about 15 mV. When the index fingernail of the tester is gently scratching on the sensor surface back and forth, the corresponding output voltage changed alternately between positive and negative (from point f to point g of the green curve in Figure 12), and its voltage amplitude is about 30 mV.

In the experiment, the time sequence data of four contact patterns, which are stroking, patting, kneading, and scratching, from the index fingers of four testers are collected, respectively. The maximum pressure of the four contact patterns applied to the sensor by each tester is less than 23 N so as to ensure that the sensor can accurately and quickly convert the pressure into voltage during the contact process. Therefore, in the data acquisition stage, even changing the shape or the size of the sensor does not affect the sequence characteristics of the four contact patterns in the time dimension. For each pattern, 75 sample sequences are collected from each tester with any of his or her index finger. Therefore, there are 300 sample sequences for each contact pattern, and a total of 1200 sample sequences are obtained for the four patterns, which are used as the dataset for the contact pattern recognition of the flexible tactile sensor based on the CNN-LSTM network.

## 4. Contact Pattern Recognition Based on the CNN-LSTM Method

In this section, by integrating the advantages of multiple single networks in feature extraction, the CNN and LSTM networks are fused in series. Then, the CNN-LSTM model with excellent expression and generalization ability is constructed to achieve the high accuracy recognition of stroking, patting, kneading, and scratching contact patterns applied to the flexible tactile sensor.

### 4.1. Principle of the CNN-LSTM Network

The CNN is a multi-layer neural network composed of convolution layers and pooling layers. It has the characteristics of local feature extraction and global feature fusion and can be well applied to the feature extraction of digital signals [36,37]. The CNN can greatly reduce the number of parameters to be optimized in the neural network through the parameter sharing mechanism of the convolution kernel. The convolutional layer of the CNN utilizes multiple different convolution kernels to obtain feature maps of multiple channels, and the feature extraction capability of the network could be improved through feature fusion of the feature maps. In the pooling layer of the CNN, the input signals are subsampled by the sliding pooling window, which reduces the dimension of the feature map while preserving local features of the network model. This paper takes advantage of CNN’s excellent spatial feature extraction ability and uses the multi-channel tactile signal from the CNN’s output as the input of the LSTM network to improve the recognition accuracy of the CNN-LSTM for the four patterns.

**Figure 13:**
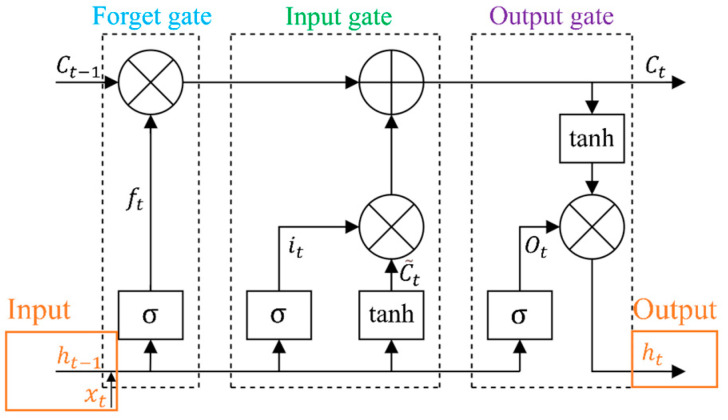
LSTM cell state at time *t*.

Based on the original recurrent neural network (RNN), LSTM neural network adds an LSTM cell (LC) structure based on forget gate (FG), input gate (IG), and output gate (OG), as shown in Figure 13. With the gated state mechanism of the LC, the problems of gradient disappearance and gradient explosion that occur in the original RNN in the process of processing long-sequence signal training were solved [38]. LSTM neural network is a temporal recurrent neural network that can be used well to mine the hidden time sequence features in long-term tactile signals [39].

In the LC state model, the input $x_{t}$ and the output information $h_{t-1}$ of the hidden layer at time t−1 are used as the input of LC at time t, and the forgetting coefficient $f_{t}$ is obtained through FG, as shown in Formula (5):

(5) $$f_{t}=\sigma \left(W_{f}\cdot \left[h_{t-1},x_{t}\right]+b_{f}\right)$$

where $W_{f}$ and $b_{f}$ are the weight matrix and bias matrix connecting the FG and input, respectively. σ is the “sigmoid” function, as shown in Formula (6):

(6) $$\sigma \left(x\right)=\frac{1}{1+\exp\left(-x\right)}$$

The degree of forgetting the historical information $C_{t-1}$ is controlled by outputting the value of the (0,1) interval through the σ function. When $f_{t}$ is 0, the historical information in LC is completely forgotten. When *f_t_* is 1, all historical information is remembered. Then, IG updates the LC state parameters $i_{t}$, ${\tilde{C}}_{t}$, and $C_{t}$ according to the input and historical information as follows:

(7) $$i_{t}=\sigma \left(W_{i}\cdot \left[h_{t-1},x_{t}\right]+b_{i}\right)$$

(8) $${\tilde{C}}_{t}=\tanh\left(W_{c}\cdot \left[h_{t-1},x_{t}\right]+b_{c}\right)$$

(9) $$C_{t}=f_{t}\cdot C_{t-1}+i_{t}\cdot {\tilde{C}}_{t}$$

where $W_{i}$ and $b_{i}$ are the weight matrix and bias matrix connecting the IG and the input, respectively. $i_{t}$ is the forgetting coefficient obtained through IG, which is used to control the forgetting degree of the current input information. $W_{c}$ and $b_{c}$ are the weight matrix and bias matrix connecting the candidate unit ${\tilde{C}}_{t}$ and the input, respectively, and ${\tilde{C}}_{t}$ is the candidate unit generated by the tanh layer. $C_{t}$ is the update result that combines the current input information and historical information. The forgetting coefficient $O_{t}$ is obtained through OG, as shown in Formula (10), which determines the degree of forgetting of $C_{t}$ at the current moment, that is, the output $h_{t}$ at time t is obtained, as shown in Formula (11):

(10) $$O_{t}=\sigma \left(W_{o}\cdot \left[h_{t-1},x_{t}\right]+b_{o}\right)$$

(11) $$h_{t}=O_{t}\cdot \tanh\left(C_{t}\right)$$

where $W_{o}$ and $b_{o}$ are the weight matrix and bias matrix connecting the OG and the input, respectively. $O_{t}$ is the forgetting coefficient obtained by OG, and $h_{t}$ is the output of the hidden layer at time t, that is, the input of the LC at time t+1.

In view of the excellent characteristics of CNN in spatial feature extraction and LSTM in processing sequence features, this paper constructs a CNN-LSTM fusion network by connecting CNN and LSTM in series to extract the features of tactile signals applied to the surface of the tactile sensor. The fully connected neural network is used to perform global feature fusion on the tactile signal features output by the LSTM network to achieve effective recognition of four contact patterns (stroking, patting, kneading, and scratching).

### 4.2. Construction of the CNN-LSTM Network for the Tactile Sensor

**Figure 14:**
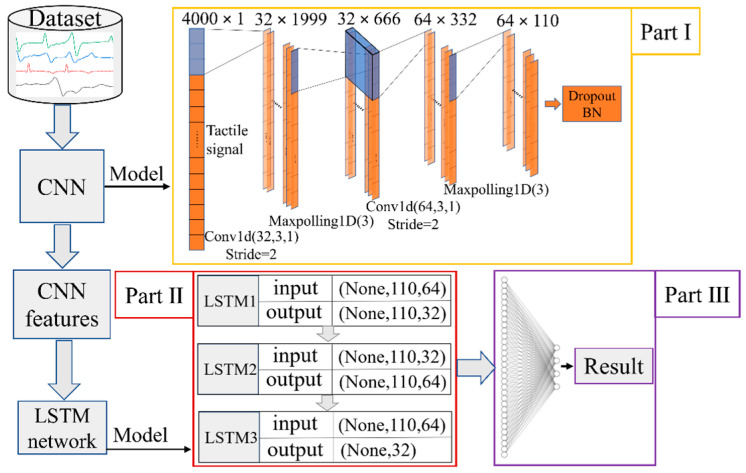
The CNN-LSTM network model for the sensor.

The CNN-LSTM network constructed in this paper is composed of three parts that are the CNN feature extraction layer, the LSTM network layer, and the fully connected classifier. Its basic model is shown in Figure 14. The input dataset of the CNN-LSTM is 1200 groups of voltage response data based on the four contact patterns collected in Section 3.5, and each group of data sample includes a 4000-dimensional time sequence signal.

The first part of the CNN-LSTM is the feature extraction layer based on CNN, which is alternately stacked by the convolution layer and the pooling layer (as shown in Figure 14). The kernel size and number of the first convolutional layer are 3 and 32, and those of the second convolutional layer are 3 and 64. Meanwhile, the ReLU is used as the activation function of the convolutional layer. The function of the convolution layer is to extract the spatial features from the 4000-dimensional tactile time sequence signals of the input data. In order to reduce the dimension of the local spatial features, the window size of the pooling layer is set to 3. The forward operation of the input tactile signals between the convolutional layer and the pooling layer can gradually extract and fuse the global spatial features of the tactile signals. The Dropout method with a regularization function is used to improve the generalization ability of the CNN-LSTM network and avoid overfitting. At the end of the CNN, the output multi-channel feature data are mapped to the standard normal distribution by the batch normalization (BN) method, which is used as the input of the LSTM network so as to improve the convergence rate of the model.

The second part of the CNN-LSTM consists of three superimposed LSTM layers which are LSTM1, LSTM2, and LSTM3 (shown in Figure 14), and their function is to extract the time dimension features of the multi-channel tactile signals that are processed by the BN method. The input of the second part is composed of 64 110-dimensional feature vectors. The output of LSTM1 contains 32 110-dimensional feature vectors, the output of LSTM2 contains 64 110-dimensional feature vectors, and the output of LSTM3 is a 32-dimensional tactile feature vector, which is the latest output of the cell state in this layer.

The third part of the CNN-LSTM consists of a fully connected neural network classifier. Its function is to fuse the 32-dimensional tactile feature vector from the second part with the four neuron nodes at the end of the model. The four neuron nodes denote a four-dimensional tactile vector from the calculation results of the CNN-LSTM model, which is mapped to the classification probability of the four contact patterns (stroking, patting, kneading, and scratching) by the Softmax function. Then, the contact pattern with the highest mapping probability is selected as the recognition result to output.

### 4.3. Result Discussion Based on the CNN-LSTM Model

**Figure 15:**
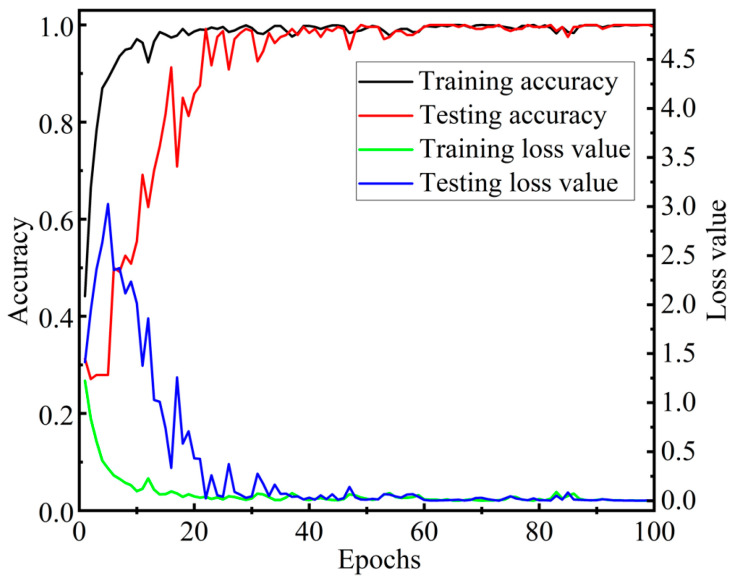
Training curves of the CNN-LSTM network.

The 1200 data samples collected from the flexible tactile sensor in Section 3.5 are randomly divided into a ratio of 8:2, and they are used as the training set and the testing set of the constructed CNN-LSTM. In the training process of the CNN-LSTM model, the multi-class cross-entropy function is used to calculate the back-propagation gradient, and the Adam algorithm is used to optimize the parameters such as the weight matrix and the bias matrix. To avoid large deviation samples appearing in the training process and to speed up the iteration rate of the network, a sample set with a batch size of 32 is used to optimize the parameters; that is, 32 samples are put into the CNN-LSTM network each time. The training set and the testing set are trained and tested for 100 iterations, respectively. Finally, the loss function value and the average accuracy of the four-contact pattern recognition for each iteration are obtained, which are shown in Figure 15.

Figure 15 implies that when the number of iterations is less than 25, the average recognition accuracy of the four contact patterns shows a rapid upward trend on the testing set. After the number of iterations is greater than 36, the average recognition accuracy of the four contact patterns is around 98%. In particular, at the 100th iteration, its average accuracy reaches 99.58%. All above indicates that the CNN-LSTM model can quickly converge to high accuracy in dealing with contact pattern recognition for the flexible tactile sensor.

**Figure 16:**
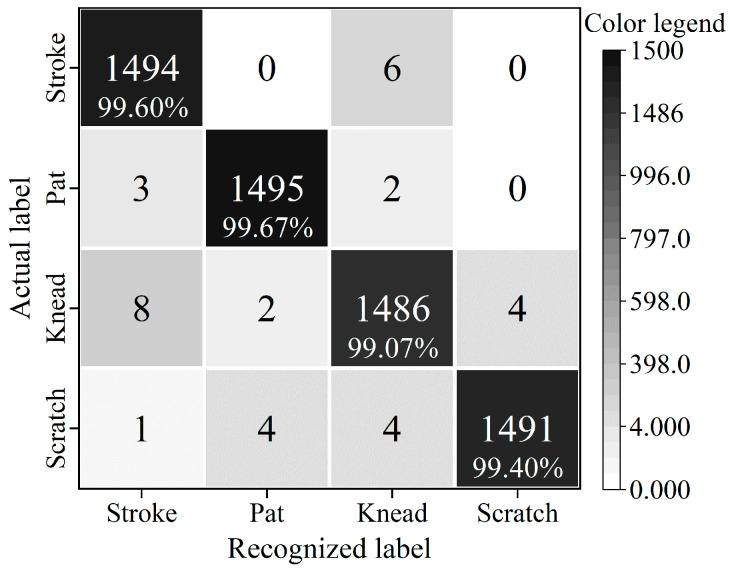
Confusion matrix of recognition results for the four contact patterns.

To precisely evaluate the accuracy of the contact pattern recognition based on the CNN-LSTM model, the 10-fold cross-validation (10-CV) method is used to construct training samples and testing samples. Firstly, the 1200 samples are randomly divided into 10 subsets without any intersection. Each subset contains 120 samples, including 30 samples from each contact pattern. Then, nine subsets are selected as the training set, and the remaining one is taken as the testing set. The procedure was repeated 10 times. It means that each subset should act as the testing set only once and should act as the training set nine times. The final result of the 10-CV is the average accuracy of the results of the 10 procedures. To make full use of the tactile signals for the 1200-time sequence, the sequence of the sample data is shuffled, and the whole 10-CV process is repeated five times. The average recognition accuracies of the four contact patterns for each 10-CV process based on the CNN-LSTM model are shown in Table 2. The corresponding confusion matrix of recognition results for the four contact patterns is shown in Figure 16, which shows the statistical results of 1500 (30×10×5) testing samples of each contact pattern from the five 10-CV processes.

Table 2 shows that the average recognition accuracy of five runs of the 10-CV method for the four contact patterns is above 99%, and their average value is 99.43%. It indicates that cross-validation is an effective method for building a training set and a testing set, and the CNN-LSTM model can recognize the four contact patterns precisely and stably. The shape or the size of the sensor may affect the sensitivity and detection limit of the sensor, which may change the output voltage of the sensor at a certain moment, but it does not significantly affect the voltage variation characteristics of the four contact patterns within a certain period, which is very important to ensure the recognition accuracy of the CNN-LSTM model. That means the shape or the size of the sensor does not obviously affect the recognition accuracy.

Figure 16 denotes that the recognition accuracies of the four contact patterns (stroking, patting, kneading, and scratching) based on the CNN-LSTM model are 99.60%, 99.67%, 99.07%, and 99.40%, respectively. In particular, the recognition accuracy of patting is higher than those of the others. The main reason is that the response voltages of stroking, kneading, and scratching alternate between positive and negative, while the response voltage of patting rises and falls rapidly, which makes it easier for the CNN-LSTM model to extract the tactile features from the patting operation. In the experiment, the response voltages of stroking alternate between positive and negative within a time span of about 200 ms (shown in Figure 12), which is almost twice as much as that of kneading and scratching. Therefore, it is easier for the CNN-LSTM model to obtain the feature of stroking in the time dimension when processing tactile sequence signals, and that makes the recognition accuracy of stroking higher than that of kneading and scratching. All of the results imply that the CNN-LSTM constructed in this paper has fairly good performance in recognizing and classifying the four contact patterns for the flexible tactile sensor.

## 5. Comparison of Recognition Based on Different Algorithms

**Figure 17:**
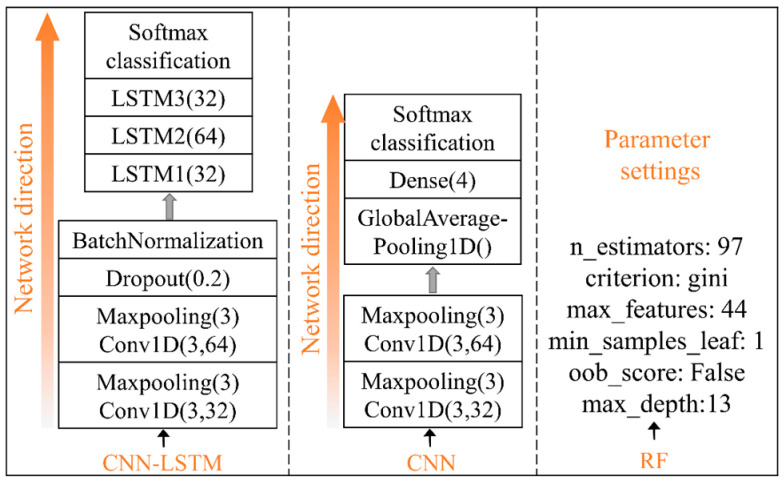
The basic structures of CNN-LSTM, CNN, and RF.

To further verify the efficiency of the CNN-LSTM model for recognizing the stroking, patting, kneading, and scratching patterns applied to the flexible tactile sensor, the CNN model and the random forest (RF) algorithm model are constructed to recognize the four contact patterns based on the same dataset as those for the CNN-LSTM model. Their basic structures are shown in Figure 17.

**Figure 18:**
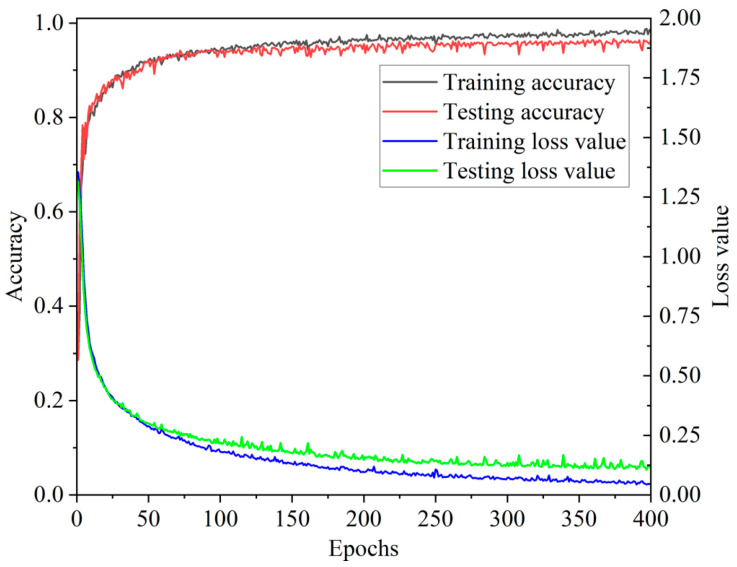
Training curves of the CNN model.

In the experiments, a fully connected neural network in the CNN model is taken to replace the three-layer LSTM network of the CNN-LSTM to fuse the extracted multi-channel tactile features. During each iteration, the multi-classification cross entropy function and Adam optimizer algorithm are used to update parameters such as the weight matrix and bias matrix of the CNN model. The input size of the batch is set to 32, and the parameters of the CNN are the same as those in the CNN-LSTM. The average recognition accuracies of the four contact patterns in the training process are shown in Figure 18. Figure 18 denotes that during the 50th to 400th iterations, the accuracy of the CNN model on the testing set is up to 96.67%, but its convergence rate is slow. Figure 15 shows that the accuracy of the CNN-LSTM fusion model exceeds 96.67% for the first time and reaches 99.17% at the 22nd iteration. It means that the performance of the CNN-LSTM for contact pattern recognition is better than that of the CNN model.

The RF algorithm selects different features from the tactile sequences to construct decision trees with different attributes, then the recognition results of all decision trees are counted, and the contact pattern with the largest number of votes is taken as the final recognition result. In the process of building the RF model, the parameters are adjusted by the grid search (GridSearchCV) method; that is, n_estimators, max_features, max_depth, and other parameters are combined for scanning, and the average recognition accuracy of the four contact patterns are used to evaluate the efficiency of the combined parameters. Finally, the combination of the optimal parameters for the RF model is determined, as shown in Figure 17.

**Figure 19:**
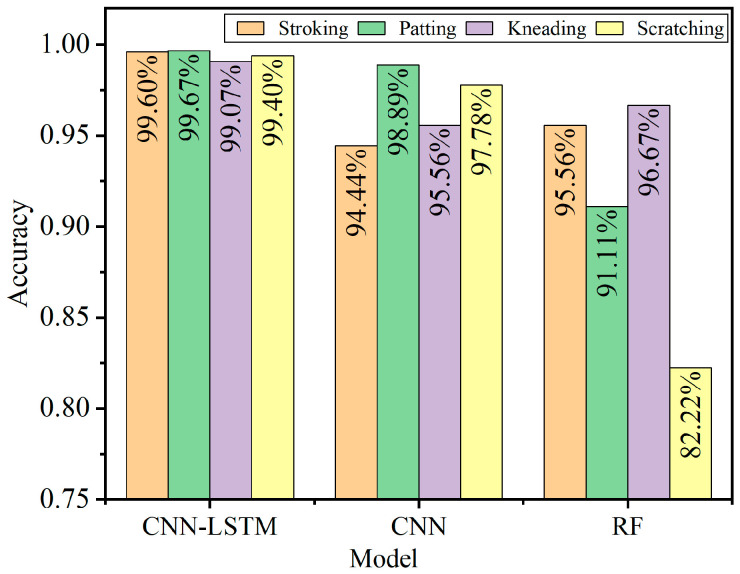
Recognition accuracies based on the three models.

The recognition accuracies of the four contact patterns based on the CNN-LSTM model, the CNN model, and the RF model are shown in Figure 19 separately. The corresponding average recognition accuracies are shown in Table 3.

Figure 19 demonstrates that recognition accuracies of the CNN-LSTM for the four contact patterns are 99.60%, 99.67%, 99.07%, and 99.40%, which are higher than those of the other two models. In addition, Table 3 implies that the average accuracies of the four contact patterns based on the three models are 99.43%, 96.67%, and 91.39%, respectively, which means that the average recognition accuracy of the CNN-LSTM is 2.76 percentage points and 8.04 percentage points higher than that of the CNN and the RF algorithm, respectively. The results also show that recognition accuracies of the models constructed based on the CNN are higher than those of the RF model, which means that the CNN model with the convolution layer and pooling layer has better robustness, and it is very suitable for processing tactile information with high-dimensional features and large capacity. The CNN-LSTM fusion model adds a three-layer LSTM behind the CNN, its convergence performance is significantly improved compared with the CNN model, and the recognition accuracies of the four contact patterns are higher than those of the CNN. Since the features of tactile signals are mainly concentrated on high-dimensional time sequences, it is difficult for the RF model to construct feature selection for decision trees when processing tactile signals, which results in relatively lower recognition accuracy. All of the results imply that the CNN-LSTM model has higher recognition accuracy than that of the CNN and RF model, and it could be well applied to the recognition of the contact patterns for the tactile sensor.

## 6. Application of the Sensor

**Figure 20:**
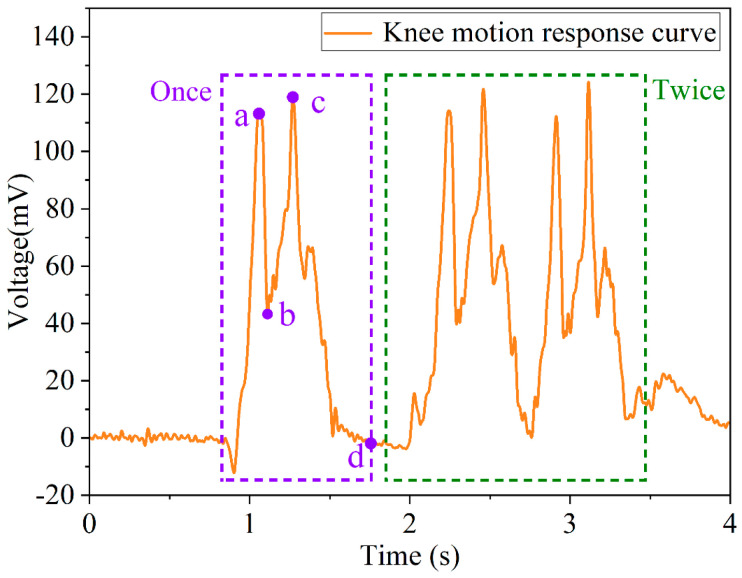
Keen bend motion detection.

The flexible tactile sensor could be applied to knee motion detection. In the experiment, the sensor is attached to the tester’s right knee, and the tester sits on a fixed seat with a height of 50 cm. At the initial state, the tester keeps his leg straight and at a 30-degree angle to the floor. There are two stages in one knee bend motion: at the first stage, the right calf of the tester should be retracted so that the angle between the right calf and the right thigh is 90-degree; at the second stage, the right calf of the tester should be straightened so that the right calf and the right thigh are in a straight line. The tester performs one knee bend motion and two consecutive knee bend motion tests, respectively, and the voltage curve of the sensor obtained is shown in Figure 20.

It can be seen from the response curve of the knee bend motion in Figure 20 (the purple dashed box) that when the tester performs the first-stage motion, the response voltage of the sensor gradually increases to point a, and the response voltage rapidly drops to point b; when the tester performs the second-stage motion, the response voltage rapidly rises from point b to point c. After completing the knee bend motion, the response voltage recovered from point c to the initial state (point d). When the tester performs two consecutive knee bend motion tests, the response voltage curve (the green dashed box in Figure 20) of the sensor is almost consistent with the variation characteristics of the one knee bend motion, which indicates that the sensor has a fast response capability and fairly good repeatability. The experimental results imply that the flexible sensor has very good application prospects in the motion state detection field.

## 7. Conclusions

A flexible tactile sensor based on PVDF material for contact pattern recognition is proposed and fabricated in this paper. The sensor has a fast response capability of 3.2 ms, which can be used to collect tactile information from different contact patterns, such as stroking, patting, kneading, and scratching. The CNN-LSTM fusion model, the CNN model, and the RF algorithm model are constructed to classify and recognize the four contact patterns, respectively. The average recognition accuracy of the four contact patterns based on the three models are 99.43%, 96.67%, and 91.39%, respectively, which means that the recognition accuracy of the CNN-LSTM is 2.76 percentage points and 8.04 percentage points higher than that of the CNN and the RF algorithm. Meanwhile, the convergence rate of the CNN-LSTM is significantly improved, which can efficiently extract complete data features from tactile signals. All of the results demonstrate that the CNN-LSTM fusion model has great global feature extraction ability and a fast convergence rate and can be very well applied to contact pattern recognition of flexible tactile sensors. The experiment results of the keen motion imply that the flexible sensor has very good application prospects in the motion state detection field.

In future work, the embedded devices will be used to deploy the CNN-LSTM model, which has been trained in this paper to realize real-time classification of different tactile signals in practical applications; we will construct a sensitive element array for the tactile sensor and make the artificial skin for robots, and the robot could correctly detect and judge the pressure with which contact pattern applied to itself and make an appropriate response.

## Figures and Tables

**Table 1:** Material properties.

Material	Density (Kg/m^3^)	Relative Permittivity	Young’s Modulus (kPa)	Poisson Ratio	e_33_ (C/m^2^)
PDMS	970	2.75	750	0.49	/
PVDF	1780	{7.4,9.3,7.6}	2.45 × 10^6^	0.4	−0.027

**Table 2:** Average accuracies of five times 10-CV method.

	1st Time	2nd Time	3rd Time	4th Time	5th Time
Average accuracy	99.50%	99.25%	99.58%	99.58%	99.25%

**Table 3:** Average recognition accuracies of the four patterns based on different models.

	CNN-LSTM	CNN	RF
Average accuracy	99.43%	96.67%	91.39%

## Data Availability

The data used to support the study are available upon request to the corresponding author.
